# Disease severity and lyso-Gb3 are associated with early echocardiographic markers of cardiac involvement in Fabry disease: a single-center observational study

**DOI:** 10.1186/s13023-026-04511-2

**Published:** 2026-07-29

**Authors:** Deniz Mitil, Zeynel Abidin Sayiner, Gökhan Altunbaş, Merve Binicier İnce, Kayahan Tekinşen, İlkay Doğan

**Affiliations:** 1 Department of Endocrinology and Metabolism, Gaziantep University Faculty of Medicine Hospital, Gaziantep, Türkiye; 2 Department of Endocrinology and Metabolism, SANKO University Faculty of Medicine Hospital, Gaziantep, Türkiye; 3 Department of Cardiology, Gaziantep University Faculty of Medicine, Gaziantep, Türkiye; 4 Department of Biostatistics, Gaziantep University, Gaziantep, Türkiye

## Abstract

**Background:**

Cardiac involvement in Fabry disease often begins with subtle functional abnormalities before overt hypertrophy or systolic dysfunction becomes evident. Identifying early echocardiographic markers associated with disease burden may improve risk stratification in this rare disorder.

**Methods:**

In this single-center cross-sectional observational study, 19 genetically confirmed Fabry disease patients receiving stable enzyme replacement therapy underwent comprehensive clinical, biochemical, and echocardiographic evaluation. Disease severity was assessed using the Mainz Severity Score Index (MSSI), and plasma lyso-globotriaosylsphingosine (Lyso-Gb3) levels were measured by LC–MS/MS. Associations between MSSI, Lyso-Gb3, and echocardiographic parameters reflecting early cardiac dysfunction were analyzed.

**Results:**

The mean MSSI score was 26.1 ± 14.2, and plasma Lyso-Gb3 levels were markedly elevated. Higher MSSI scores were associated with increased E/e′ septal (*r* = 0.52, *p* = 0.021), E/e′ average (*r* = 0.51, *p* = 0.027), and tricuspid regurgitation velocity (*r* = 0.60, *p* = 0.006). Plasma Lyso-Gb3 levels correlated positively with left ventricular mass index (*r* = 0.60, *p* = 0.031), posterior wall thickness (*r* = 0.62, *p* = 0.023), and tricuspid regurgitation velocity (*r* = 0.80, *p* = 0.001), and negatively with left ventricular ejection fraction (*r* = − 0.72, *p* = 0.005). These associations were observed despite preserved systolic function in most patients.

**Conclusions:**

Disease severity assessed by MSSI and circulating Lyso-Gb3 levels are associated with early functional and structural echocardiographic abnormalities in Fabry disease. Incorporating these measures into routine evaluation may facilitate earlier identification of cardiac involvement and support individualized monitoring strategies in this rare disease population.

## Introduction

Fabry disease is a rare, X-linked lysosomal storage disorder caused by mutations in the GLA gene, which lead to deficient or absent α-galactosidase A (α-Gal A) activity. This enzymatic deficiency results in the progressive accumulation of globotriaosylceramide (Gb3) and its deacylated derivative, globotriaosylsphingosine (Lyso-Gb3), in various tissues including the heart, kidneys, and nervous system [1]. Cardiac involvement is one of the most prevalent and life-threatening manifestations of Fabry disease and encompasses a spectrum ranging from left ventricular hypertrophy (LVH) to arrhythmias, conduction abnormalities, and ultimately heart failure [2]. Recent evidence suggests that cardiac dysfunction in Fabry disease often begins in a subclinical phase, characterized by impaired diastolic function and myocardial remodeling before overt systolic decline is detectable [3]. Advanced echocardiographic techniques offer critical insights into the myocardial changes that occur in Fabry disease. Tissue Doppler imaging (TDI) allows for the quantification of myocardial velocities and estimation of filling pressures, which are essential for identifying diastolic dysfunction [4]. An elevated E/e′ ratio, reflecting increased left ventricular filling pressures, has been proposed as a sensitive marker of subclinical myocardial involvement. In parallel, left ventricular mass index (LVMI) and interventricular septal thickness, both of which are often increased in Fabry patients, represent structural adaptations associated with progressive glycosphingolipid deposition [5]. Notably, these alterations may develop in the presence of preserved ejection fraction, underscoring the need for early functional assessment. The integration of these parameters, including TR velocity and left atrial volume index (LAVI), provides a comprehensive framework for the early detection and monitoring of Fabry cardiomyopathy [6]. Lyso-Gb3, a circulating biomarker reflecting disease burden, has been shown to correlate with phenotype severity and organ involvement [7]. However, its relationship with echocardiographic indices of cardiac dysfunction particularly those signaling subclinical disease remains incompletely defined. Moreover, the Mainz Severity Score Index (MSSI), a validated clinical tool encompassing general, renal, cardiac, and neurologic components, provides a composite measure of disease burden but its value in predicting cardiac involvement has not been fully elucidated [8]. The aim of this study was to investigate how disease severity, assessed by the Mainz Severity Score Index, and circulating Lyso-Gb3 levels relate to early structural and functional cardiac alterations in Fabry disease, with emphasis on echocardiographic parameters that reflect early myocardial involvement and functional impairment.

## Materials and methods

### Study design and participants

This was a single-center cross-sectional observational study conducted at the Department of Endocrinology and Metabolism. All participants underwent a single clinical, biochemical, and echocardiographic assessment during the study period, and no longitudinal follow-up measurements were included in the present analysis. Nineteen adult patients (≥ 18 years) with genetically confirmed Fabry disease (13 males, 6 females) were consecutively enrolled. All participants were receiving a stable enzyme replacement therapy (ERT) regimen for at least 12 months and were under regular follow-up for a minimum of six months prior to inclusion.

Fabry disease diagnosis was established based on reduced or absent α-galactosidase A (α-Gal A) activity in plasma and/or leukocytes, together with the identification of pathogenic or likely pathogenic variants in the *GLA* gene using Sanger sequencing. All patients underwent clinical evaluation, laboratory testing, and transthoracic echocardiography within a one-month interval to ensure temporal consistency of assessments.

Exclusion criteria included significant cardiac disease unrelated to Fabry disease (such as severe valvular disease or ischemic cardiomyopathy), previous myocardial infarction or coronary revascularization, uncontrolled hypertension (systolic blood pressure ≥ 160 mmHg or diastolic ≥ 100 mmHg), diabetes mellitus with established end-organ damage, coexisting inflammatory or infiltrative disorders (e.g., amyloidosis or sarcoidosis), inadequate echocardiographic image quality, pregnancy or lactation, and participation in investigational drug trials within the preceding three months.

### Clinical assessment and disease severity

Disease severity was assessed using the Mainz Severity Score Index (MSSI), a validated composite scoring system that evaluates general, neurological, renal, and cardiovascular involvement. Total MSSI scores were categorized as mild (< 20), moderate (20–40), or severe (> 40). All clinical and biochemical data were recorded during the same visit.

### Echocardiographic assessment

Comprehensive transthoracic echocardiography was performed using a Philips EPIQ 7 ultrasound system (Philips Healthcare, Andover, MA, USA) equipped with an X5-1 transducer. Examinations were conducted with patients in the left lateral decubitus position by an experienced cardiologist blinded to clinical and laboratory data, in accordance with current American Society of Echocardiography guidelines.

Standard two-dimensional and Doppler measurements included interventricular septal thickness, posterior wall thickness, left ventricular mass index (LVMI) calculated using the Devereux formula, left atrial volume index (LAVI) assessed by the area–length method, left ventricular ejection fraction (LVEF) measured using the modified Simpson’s method, and tricuspid regurgitation velocity obtained by continuous-wave Doppler. Tissue Doppler imaging was used to measure septal and lateral mitral annular velocities, including early diastolic (e′), late diastolic (a′), and systolic (s′) velocities. E/e′ ratios (septal, lateral, and average) were calculated to estimate left ventricular filling pressures.

### Biochemical and genetic analyses

Fasting venous blood samples were collected in EDTA tubes and processed by centrifugation at 3000 rpm for 10 min. Plasma samples were stored at − 80 °C until analysis. Plasma lyso-globotriaosylsphingosine (Lyso-Gb3) levels were quantified using liquid chromatography–tandem mass spectrometry (LC–MS/MS), and all Lyso-Gb3 results are reported in ng/mL throughout the manuscript. α-Gal A activity was measured using a fluorometric assay. Genetic analysis of the *GLA* gene was performed by Sanger sequencing.

### Statistical analysis

Continuous variables were expressed as mean ± standard deviation or median (range), as appropriate, and categorical variables as frequencies and percentages. Data distribution was assessed using the Shapiro–Wilk test. Comparisons across MSSI severity categories were performed using the Kruskal–Wallis test. Associations between continuous variables were analyzed using Spearman’s rank correlation coefficient. A two-sided p value < 0.05 was considered statistically significant. Statistical analyses were conducted using IBM SPSS Statistics for Windows, version 22.0 (IBM Corp., Armonk, NY, USA).

## Results

A total of 19 genetically confirmed Fabry patients (13 males, 6 females; mean age 40.2 ± 12.0 years) were included in the study. Plasma globotriaosylsphingosine (Lyso-Gb3) levels were markedly elevated in the study cohort (32.55 ± 31.34 ng/mL). The mean α-galactosidase A (α-Gal A) enzyme activity was 1.55 ± 1.93 µmol/L/h (reference range: 4–20 µmol/L/h). The mean Mainz Severity Score Index (MSSI) score was 26.1 ± 14.2, with disease severity categorized as mild (*n* = 7), moderate (*n* = 6), and severe (*n* = 6). Mean BMI was 23.9 ± 5.2 kg/m². Renal parameters showed a mean glomerular filtration rate (GFR) of 75.0 ± 48.4 mL/min/1.73 m² and proteinuria of 543.9 ± 566.6 mg/day. Mean enzyme replacement therapy (ERT) duration was 8.4 ± 2.7 years. (Table 1)

**Table 1:** Demographic, clinical, genetic, and biochemical characteristics of Fabry patients (*n* = 19)

Variable	Result
Age (years)	40.2 ± 12.0
Sex (n, %)	Male: 13 (68%), Female: 6 (32%)
BMI (kg/m²)	23.9 ± 5.2
α-Gal A enzyme (µmol/L/h)	1.55 ± 1.93
Lyso-Gb3 (ng/mL)	32.55 ± 31.34
MSSI score	26.1 ± 14.2
ERT duration (years)	8.4 ± 2.7
Severity classification	Mild: 7, Moderate: 6, Severe: 6
Mutations	Most frequent: c.851T > C (*n* = 6), c.679 C > T (*n* = 4), c.376 A > G (*n* = 4); others rare (*n* = 1 each)
GFR (mL/min/1.73 m²)	75.0 ± 48.4

Group comparisons demonstrated progressive structural and functional impairment with increasing disease severity. Left ventricular ejection fraction demonstrated a trend toward lower values with increasing disease severity (59.0 ± 3.37% in the mild group versus 55.83 ± 2.04% in the severe group, *p* = 0.058). Interventricular septal thickness (IVS) and posterior wall thickness (PW) showed numerical increases, although not statistically significant (*p* = 0.237 and *p* = 0.235, respectively).Early diastolicmitral inflow velocity (E) was (0.72 ± 0.09 vs. 0.72 ± 0.12 vs. 0.97 ± 0.29; *p* = 0.070) across mild, moderate, and severe groups, respectively. Late diastolic atrial contraction velocity (A) measured (0.83 ± 0.06 vs. 0.77 ± 0.11 vs. 0.85 ± 0.30; *p* = 0.515). Correspondingly, the E/A ratio increased across disease severity, with values of (0.87 ± 0.12 vs. 0.94 ± 0.19 vs. 1.22 ± 0.49; *p* = 0.060). E/e′ septal increased significantly among mild, moderate, and severe groups (11.51 ± 2.81 vs. 13.58 ± 3.50 vs. 23.71 ± 11.10; *p* = 0.042). Similarly, E/e′ lateral showed a similar pattern of elevation (9.17 ± 2.74 vs. 12.14 ± 5.18 vs. 21.86 ± 9.80; *p* = 0.048). A trend toward higher tricuspid regurgitation velocity was observed across disease severity groups, increasing from 1.64 ± 0.57 m/s in the mild group to 2.37 ± 0.30 m/s in the severe group, although statistical significance was not reached (*p* = 0.058). A trend toward increasing left ventricular mass index (LVMI) was observed across disease severity groups (107.55 ± 17.81 vs. 139.34 ± 28.52 vs. 149.14 ± 45.97 g/m², *p* = 0.061). Left atrial volume index (LAVI), LA diameter, and left ventricular mass also showed numerical increases across groups; however, these differences did not reach statistical significance (all *p* > 0.05) (Table 2).

**Table 2:** Echocardiographic parameters according to MSSI severity category

	Mild	Moderate	Severe	*p* value
IVS (cm)	1,16 ± 0,19	1,4 ± 0,27	1,35 ± 0,19	0.237
LVEDD (cm)	4,77 ± 0,27	4,5 ± 0,45	4,82 ± 0,77	0.490
PW (cm)	1,11 ± 0,13	1,25 ± 0,08	1,17 ± 0,32	0.235
LA diameter (cm)	3.63 ± 0.23	3.80 ± 0.62	3.73 ± 0.54	0.814
LVEF (%)	59 ± 3,37	58,83 ± 2,04	55,83 ± 2,04	0.058
Mitral E velocity (m/s)	0,72 ± 0,09	0,72 ± 0,12	0,97 ± 0,29	0.070
Mitral A velocity (m/s)	0,83 ± 0,06	0,77 ± 0,11	0,85 ± 0,3	0.515
E/A	0,87 ± 0,12	0,94 ± 0,19	1,22 ± 0,49	0.060
TDI Septal e’	0,07 ± 0,02	0,06 ± 0,01	0,12 ± 0,17	0.510
TDI Lateral e’	0,08 ± 0,02	0,06 ± 0,02	0,1 ± 0,12	0.230
E/e’ Septal	11,51 ± 2,81	13,58 ± 3,5	23,71 ± 11,1	**0.042**
E/e’ Lateral	9,17 ± 2,74	12,14 ± 5,18	21,86 ± 9,8	**0.048**
E/e′ Average	10,34 ± 2,76	12,86 ± 3,35	22,78 ± 8,6	**0.044**
TR Velocity (m/s)	1,64 ± 0,57	2,27 ± 0,52	2,37 ± 0,3	0.058
LAV (mL)	50,98 ± 18,77	55,18 ± 28,32	62,4 ± 24,44	0.728
LAVI (mL/m²)	29,03 ± 8,32	32,72 ± 15,19	37,75 ± 6,49	0.355
LVM (g)	185,91 ± 35,69	232,06 ± 60,67	247,37 ± 114,16	0.344
LVMI (g/ m²)	107,55 ± 17,81	139,34 ± 28,52	149,14 ± 45,97	0.061

Values are presented as mean ± SD. Comparisons between groups were performed using the Kruskal–Wallis test

Lyso-Gb3 levels showed a strong negative correlation with ejection fraction (*r* = − 0.72, *p* = 0.005), a strong positive correlation with LVMI (*r* = 0.60, *p* = 0.031), a strong positive correlation with posterior wall thickness (*r* = 0.62, *p* = 0.023), and a very strong positive correlation with TR velocity (*r* = 0.80, *p* = 0.001). MSSI scores demonstrated a moderate positive correlation with E/e′ septal (*r* = 0.52, *p* = 0.021), a moderate positive correlation with E/e′ average (*r* = 0.51, *p* = 0.027), and a strong positive correlation with TR velocity (*r* = 0.60, *p* = 0.006). α-Gal A activity showed a moderate positive correlation with ejection fraction (*r* = 0.59, *p* = 0.008), a moderate negative correlation with LVMI (*r* = − 0.49, *p* = 0.031), and a moderate negative correlation with TR velocity (*r* = − 0.51, *p* = 0.027) (Table 3).

**Table 3:** Spearman correlations between lyso-Gb3, MSSI score, α-Galactosidase A Activity, and echocardiographic parameters

Parameter	Lyso-Gb3	MSSI score	α-Gal A activity
LVEF (%)	-0.72 **(*p* = 0.005)**	-0.54 **(*p* = 0.017)**	0.59 **(*p* = 0.008)**
LVMI (g/m²)	0.60 **(*p* = 0.031)**	0.61 **(*p* = 0.005)**	-0.49 **(*p* = 0.031)**
TR velocity (m/s)	0.80 **(*p* = 0.001)**	0.60 **(*p* = 0.006)**	-0.51 **(*p* = 0.027)**
E/e’ Septal	0.16 (*p* = 0.596)	0.52 **(*p* = 0.021)**	-0.44 (*p* = 0.062)
E/e’ Average	0.16 (*p* = 0.603)	0.51 **(*p* = 0.027)**	-0.48 **(*p* = 0.038)**
Posterior wall thickness (cm)	0.62 **(*p* = 0.023)**	0.17 (*p* = 0.499)	-0.12 (*p* = 0.611)

Values are presented as Spearman correlation coefficients (r) with corresponding p values

## Discussion

Cardiac involvement is a major determinant of morbidity and mortality in Fabry disease and may remain clinically silent for years before overt hypertrophy, systolic dysfunction, or heart failure becomes apparent [9, 10]. Early identification of myocardial involvement is therefore critical, particularly in the era of disease-specific therapies. In this study, we found that both global disease severity, assessed by the Mainz Severity Score Index (MSSI), and circulating Lyso-Gb3 levels were associated with early echocardiographic markers of cardiac dysfunction, even in patients with largely preserved left ventricular ejection fraction.

A key finding of our study is the consistent association between increasing MSSI scores and echocardiographic indices of diastolic dysfunction, particularly the E/e′ ratio and tricuspid regurgitation velocity. These parameters reflect elevated left ventricular filling pressures and early hemodynamic consequences of myocardial involvement. Importantly, these associations were observed before marked systolic impairment, supporting the concept that Fabry cardiomyopathy initially evolves through functional and microstructural alterations rather than overt contractile failure [3, 5]. Previous studies have shown that diastolic abnormalities and increased filling pressures precede left ventricular hypertrophy and are associated with myocardial fibrosis detected by cardiac magnetic resonance imaging [3, 5, 11, 12].

Lyso-Gb3 has emerged as a central biomarker in Fabry disease, reflecting substrate accumulation, disease activity, and phenotype severity [7, 13]. In the present study, Lyso-Gb3 levels showed strong correlations with both structural and functional echocardiographic parameters, including left ventricular mass index, posterior wall thickness, tricuspid regurgitation velocity, and ejection fraction. These associations persisted despite long-term enzyme replacement therapy, suggesting that Lyso-Gb3 may capture residual disease burden and ongoing pathophysiological processes not fully reversed by treatment [14–18]. This observation is consistent with growing evidence that chronic inflammatory signaling, microvascular dysfunction, and interstitial remodeling continue to contribute to cardiac disease progression in Fabry patients receiving enzyme replacement therapy [16, 17].

Previous studies have demonstrated that myocardial involvement in Fabry disease may begin before the development of overt left ventricular hypertrophy [3, 5, 11, 12]. Advanced imaging modalities, particularly cardiac magnetic resonance (CMR), have provided important insights into myocardial storage, fibrosis, and tissue remodeling in Fabry disease. Native T1 mapping and late gadolinium enhancement have emerged as valuable tools for the detection of early myocardial involvement and risk stratification [11, 12]. Although CMR was not available in the present study, the observed associations between MSSI, Lyso-Gb3, and echocardiographic parameters are compatible with the concept that subclinical cardiac involvement may already be present before overt functional deterioration becomes apparent. Furthermore, while CMR offers superior myocardial tissue characterization, its routine use may be limited by availability, accessibility, and cost considerations. Therefore, readily available echocardiographic parameters remain clinically relevant for routine cardiac assessment and longitudinal follow-up in Fabry disease.

### Novelty and clinical implications

The novel contribution of this study lies in the integrated evaluation of a validated clinical severity score (MSSI), a disease-specific biochemical marker (Lyso-Gb3), and echocardiographic indices reflecting early cardiac involvement. While previous studies have independently examined echocardiographic findings or biomarker levels in Fabry disease, data linking these domains in a cohesive manner remain limited [7, 8, 13]. Our findings suggest that combining MSSI and Lyso-Gb3 assessment with routine echocardiography may enhance early detection of cardiac involvement and provide a more comprehensive representation of disease burden.

From a clinical perspective, this integrated approach may be particularly valuable for identifying patients at risk of progressive cardiac disease despite preserved systolic function. Early recognition of diastolic dysfunction and subtle structural remodeling may inform closer surveillance, prompt referral for advanced imaging such as cardiac magnetic resonance, and support individualized therapeutic decision-making [9, 19, 20].

MSSI and Lyso-Gb3 appear to provide complementary information: MSSI reflects cumulative multisystem disease burden [8, 21], whereas Lyso-Gb3 serves as a dynamic biochemical surrogate of substrate accumulation and ongoing disease activity [7, 13–15]. The observed associations between these measures and echocardiographic markers underscore their potential role in risk stratification beyond traditional parameters such as left ventricular wall thickness alone.

### Study limitations

Several limitations should be acknowledged. First, this was a single-center study with a relatively small sample size, which may limit statistical power and generalizability. Second, the cross-sectional design precludes assessment of temporal relationships and does not permit causal inference regarding the associations observed between disease severity, Lyso-Gb3 levels, and echocardiographic parameters. Third, longitudinal changes in Lyso-Gb3 concentrations and cardiac function could not be evaluated because each participant underwent only a single assessment. Fourth, all included patients were receiving stable enzyme replacement therapy, and therefore a treatment-naïve comparison group was not available. Finally, cardiac magnetic resonance imaging data were not available, preventing direct correlation of echocardiographic findings with myocardial fibrosis, native T1 values, extracellular volume measurements, or late gadolinium enhancement. Future multicenter prospective studies incorporating larger patient populations, treatment-naïve individuals, longitudinal follow-up, and advanced cardiac imaging modalities are warranted to validate and extend our findings.

## Conclusions

Disease severity assessed by MSSI and circulating Lyso-Gb3 levels were associated with early functional and structural echocardiographic abnormalities in Fabry disease. These findings support the complementary use of clinical severity assessment, biochemical biomarkers, and routine echocardiography for the early identification of cardiac involvement. Although larger longitudinal studies are needed, the integration of these readily available tools may contribute to improved risk stratification and individualized monitoring of patients with Fabry disease.

## Data Availability

The datasets used and/or analysed during the current study are available from the corresponding author on reasonable request.

## References

[1] Kok K, Zwiers KC, Boot RG, Overkleeft HS, Aerts JMFG, Artola M. Fabry Disease: Molecular Basis, Pathophysiology, Diagnostics and Potential Therapeutic Directions. Biomolecules. 2021;11(2):271. 10.3390/biom11020271 PMC7918333 33673160

[2] Azevedo O, Cordeiro F, Gago MF, Miltenberger-Miltenyi G, Ferreira C, Sousa N, Cunha D. Fabry disease and the heart: a comprehensive review. Int J Mol Sci. 2021 Apr 23;22(9):4434. 10.3390/ijms22094434 PMC8123068 33922740

[3] Augusto JB, Johner N, Shah D, Nordin S, Knott KD, Rosmini S, Lau C, Alfarih M, Hughes R, Seraphim A, Vijapurapu R, Bhuva A, Lin L, Ojrzyńska N, Geberhiwot T, Captur G, Ramaswami U, Steeds RP, Kozor R, Hughes D, Moon JC, Namdar M. The myocardial phenotype of Fabry disease pre-hypertrophy and pre-detectable storage. Eur Heart J Cardiovasc Imaging. 2021;22(7):790–9. 10.1093/ehjci/jeaa101 PMC8219366 32514567

[4] Lillo R, Pieroni M, Camporeale A, Ciabatti M, Lombardo A, Massetti M, Graziani F. Echocardiography in Anderson-Fabry Disease. Rev Cardiovasc Med. 2022;23(6):201. 10.31083/j.rcm2306201 PMC11274060 39077168

[5] Liu D, Oder D, Salinger T, Hu K, Müntze J, Weidemann F, Herrmann S, Ertl G, Wanner C, Frantz S, Störk S, Nordbeck P. Association and diagnostic utility of diastolic dysfunction and myocardial fibrosis in patients with Fabry disease. Open Heart. 2018;5(2):e000803. 10.1136/openhrt-2018-000803 PMC6045729 30018776

[6] Halfmann MC, Altmann S, Schoepf UJ, Reichardt C, Hennermann JB, Kreitner KF, Kloeckner R, Hahn F, Dueber C, Varga-Szemes A, Kampmann C, Emrich T. Left atrial strain correlates with severity of cardiac involvement in Anderson-Fabry disease. Eur Radiol. 2023 Mar;33(3):2039–51. 10.1007/s00330-022-09183-7 PMC9935647 36322192

[7] Nowak A, Mechtler TP, Hornemann T, Gawinecka J, Theswet E, Hilz MJ, Kasper DC. Genotype, phenotype and disease severity reflected by serum LysoGb3 levels in patients with Fabry disease. Mol Genet Metab. 2018;123(2):148–53. 10.1016/j.ymgme.2017.07.002 28728877

[8] Beck M. The Mainz Severity Score Index (MSSI): development and validation of a system for scoring the signs and symptoms of Fabry disease. Acta Paediatr Suppl. 2006 Apr;95(451):43–6. 10.1111/j.1651-2227.2006.tb02388.x 16720464

[9] Marek J, Chocholová B, Rob D, Paleček T, Mašek M, Dostálová G, Linhart A. Three- dimensional echocardiographic left ventricular strain analysis in Fabry disease: correlation with heart failure severity, myocardial scar, and impact on long-term prognosis. Eur Heart J Cardiovasc Imaging. 2023;24(12):1629–37. 10.1093/ehjci/jead121 PMC10667034 37309820

[10] Paelinck BP, Bondue A, Robyns T, Eyskens F. Left ventricular hypertrophy: do not forget Fabry disease. Diagnostic work-up and differential diagnosis. Acta Cardiol. 2024 Aug;79(6):642–9. 10.1080/00015385.2024.2346873 38869089

[11] Deraz SE, Esmat OD, El-Hmid RGA, Amin SA. Evaluation of diastolic dysfunction in children with hypertrophic cardiomyopathy and its relationship with development of myocardial fibrosis. Egypt Heart J. 2023;75(1):54. 10.1186/s43044-023-00382-1 PMC10313626 37389694

[12] Quail M, Grunseich K, Baldassarre LA, Mojibian H, Marieb MA, Cornfeld D, Soufer A, Sinusas AJ, Peters DC. Prognostic and functional implications of left atrial late gadolinium enhancement cardiovascular magnetic resonance. J Cardiovasc Magn Reson. 2019 Jan;3(1):2. 10.1186/s12968-018-0514-3 PMC6317232 30602395

[13] Ramaswami U, West ML, Tylee K, Castillon G, Braun A, Ren M, Doobaree IU, Howitt H, Nowak A. The use and performance of lyso-Gb3 for the diagnosis and monitoring of Fabry disease: A systematic literature review. Mol Genet Metab. 2025;145(2):109110. 10.1016/j.ymgme.2025.109110 40328031

[14] Sakuraba H, Togawa T, Tsukimura T, Kato H. Plasma lyso-Gb3: a biomarker for monitoring fabry patients during enzyme replacement therapy. Clin Exp Nephrol. 2018 Aug;22(4):843–9. 10.1007/s10157-017-1525-3 29288396

[15] Nowak A, Beuschlein F, Sivasubramaniam V, Kasper D, Warnock DG. Lyso-Gb3 associates with adverse long-term outcome in patients with Fabry disease. J Med Genet. 2022 Mar;59(3):287–93. 10.1136/jmedgenet-2020-107338. PMC8867289 33495303

[16] Biddeci G, Spinelli G, Colomba P, Duro G, Giacalone I, Di Blasi F. Fabry disease beyond storage: the role of inflammation in disease progression. Int J Mol Sci. 2025 Jul 22;26(15):7054. 10.3390/ijms26157054 PMC12346724 40806187

[17] Nowak A, Arends M, van der Tol L, Weidemann F, Wijburg FA, Mehta A, et al. Plasma Lyso-Gb3 predicts clinical events and disease progression in Fabry disease. J Med Genet. 2023;60(8):731–9.

[18] McCarron EP, Chinnadurai R, Meyer J, Anderson T, Stepien KM, Sharma R, Woolfson P, Jovanovic A. Real-world clinical outcomes in adult patients with Fabry disease: a 20-year retrospective observational cohort study from a single centre. Mol Genet Metab Rep. 2025 May;14:43:101229. 10.1016/j.ymgmr.2025.101229 PMC12144493 40485669

[19] Moura B, Aimo A, Al-Mohammad A, Flammer A, Barberis V, Bayes-Genis A, et al. Integration of imaging and circulating biomarkers in heart failure: a consensus document by the Biomarkers and Imaging Study Groups of the Heart Failure Association of the European Society of Cardiology. Eur J Heart Fail. 2021;23(10):1577–96. 10.1002/ejhf.2339 34482622

[20] Hesse K, Khanji MY, Aung N, Dabbagh GS, Petersen SE, Chahal CAA. Assessing heterogeneity on cardiovascular magnetic resonance imaging: a novel approach to diagnosis and risk stratification in cardiac diseases. Eur Heart J Cardiovasc Imaging. 2024 Mar 27;25(4):437–45. 10.1093/ehjci/jead285 PMC10966332 37982176

[21] Whybra C, Kampmann C, Krummenauer F, Ries M, Mengel E, Miebach E, Baehner F, Kim K, Bajbouj M, Schwarting A, Gal A, Beck M. The Mainz Severity Score Index: a new instrument for quantifying the Anderson-Fabry disease phenotype, and the response of patients to enzyme replacement therapy. Clin Genet. 2004;65(4):299–307. 10.1111/j.1399-0004.2004.00219.x 15025723

